# Superior properties in room-temperature colloidal-dot quantum emitters revealed by ultralow-dark-count detections of temporally-purified single photons

**DOI:** 10.1038/s41598-019-52377-1

**Published:** 2019-11-04

**Authors:** Toshiyuki Ihara, Shigehito Miki, Toshiki Yamada, Takahiro Kaji, Akira Otomo, Iwao Hosako, Hirotaka Terai

**Affiliations:** 1 Advanced ICT Research Institute, National Institute of Information and Communications Technology, 588-2, Iwaoka, Nishi-ku, Kobe, Hyogo, 651-2492 Japan; 2 Kobe University, 1-1, Rokkodai-cho, Nada-ku, Kobe, Hyogo, 657-8501 Japan

**Keywords:** Quantum dots, Single photons and quantum effects

## Abstract

The realization of high-quality quantum emitters that can operate at room temperature is important for accelerating the application of quantum technologies, such as quantum communication, quantum information processing, and quantum metrology. In this work, we study the photon-antibunching properties on room-temperature emission from individual colloidal quantum dots (CQDs) using superconducting-nanowire single-photon detectors and temporal filtering of the photoluminescence decay curve. We find that high single-photon purities and high photon-generation rates can be simultaneously achieved by removing the signals originating from the sequential two-photon emission of biexcitons created by multiple excitation pulses. We successfully demonstrate that the ultrahigh performance of the room-temperature single-photon sources showing g^(2)^(0) ≪ 10^−2^ can be confirmed by the ultralow-dark-count detection of the temporally purified single photons. These findings provide strong evidence for the attractiveness of CQDs as candidates for high-quality room-temperature quantum light sources.

## Introduction

The characteristics of solid-state quantum light sources, such as single-photon sources (SPSs) that can generate antibunched photons, have been extensively studied for more than 15 years^1–8^. Such research has attracted attention because SPSs can be applied in various quantum technologies such as quantum cryptography^9^, quantum computing^10^, quantum metrology^11^, and quantum imaging^12^. In the last decade, room-temperature generation of single photons in the visible region has been studied with various materials and devices^13–29^. The realization of high-quality room-temperature SPSs in the visible region is expected to trigger acceleration of the applicability of quantum technologies. However, the performance of room-temperature SPSs is not very high compared to cryogenic SPSs^30–35^. In particular, the purity of single photons, determined by the photon correlation value g^(2)^ at the time origin g^(2)^(0), is often poor. While advanced applications of quantum light sources require high single-photon purity of g^(2)^(0) < 0.01^7^, actual room-temperature SPSs have shown g^(2)^(0) values of 0.02 to 0.5^13–29^. The main reason underlying this problem is the large spectral overlap of two photons emitted sequentially from a single system, which causes difficulties in spectroscopic single-photon purification. At cryogenic temperatures, the single-photon purity could be easily improved by separating the two photons based on their difference in emission wavelength^30–35^.

In recent years, the above problem of poor purity in room-temperature SPSs has been partially solved by the application of temporal filtering to the photoluminescence (PL) decay curves^36–42^. This technique of temporal single-photon purification is very effective for systems having sequential two-photon emission (STPE) with different PL lifetimes. One such system is that of colloidal quantum dots (CQDs), where the PL lifetimes of the STPE process of two electron–hole pairs (biexcitons) are usually different^40,41^. CQDs should be attractive materials for room-temperature SPSs, because they have high PL efficiencies covering almost all ranges in the visible region. Recent reports have indicated, even without the use of temporal filters, the realization of high purities showing g^(2)^(0) ranging from 0.03 to 0.1 from various CQDs^18,19,25,27^. Therefore, it is expected that the use of temporal filtering facilitates the realization of higher single-photon purities showing g^(2)^(0) of ≪0.01. However, even if temporal purification is used, another problem impedes observation of the small g^(2)^(0) value: a background offset appears in the g^(2)^ curve, caused by the dark counts of the single-photon detector. This background offset in the g^(2)^ curve must be solved for successful experiments in the visible region, because conventional detectors commonly used for visible-region photons, such as avalanche photodiodes and photomultipliers, have large dark counts. This issue can be addressed by using superconducting-nanowire single-photon detectors (SSPDs), whose dark counts are much smaller than those of conventional detectors. While SSPDs are usually designed for the infrared region, we recently reported an SSPD design with high detection efficiencies in the visible region^43^. By using such SSPDs, the properties of room-temperature SPSs, which are easily obscured by the dark counts of detectors, may be revealed.

In the present study, we report successful observations of superior properties in room-temperature SPSs comprising CQDs, which are revealed by the use of SSPDs and temporal filtering. One of the revealed properties is that, by removing the signals originating from the STPE of biexcitons created by not only single pulse but also multiple excitation pulses, high single-photon purities can be achieved simultaneously with high photon-generation rates. We experimentally demonstrate that the use of the temporal filter enables a significant improvement in the single-photon purity by a factor of 20, resulting in the autocorrelation signal showing g^(2)^(0) = 0.005. We numerically show that the ultralow dark-count detection of the temporally purified single photons facilitates the observation of ultrahigh performance in room-temperature SPSs showing g^(2)^(0) of <10^−5^.

## Results

### g^(2)^ dip structure revealed by a temporal filter

**Figure 1:**
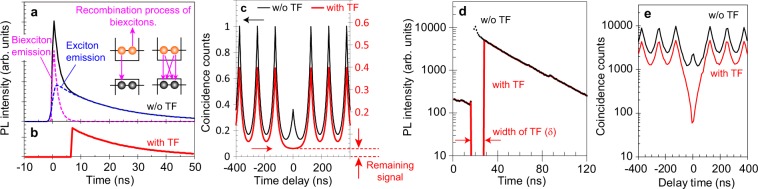
g^(2)^ analysis based on a use of temporal filter. (**a**) Calculated photoluminescence (PL) decay curve without temporal filter (TF). The total PL decay is composed of biexciton PL (pink) and exciton PL (blue). (**b**) Calculated PL decay curve with TF. (**c**) The calculated g^(2)^ curves without (black) and with (red) applications of TF. (**d**) Experimental data of PL decay curves without (black) and with (red) applications of TF obtained from Sample A. (**e**) Corresponding data of g^(2)^ curves without (black) and with (red) applications of TF. The application of TF causes a significant change in the g^(2)^ line shape around the time origin from a peak to a dip structure.

In Fig. 1a–c, we show the calculated PL decay and g^(2)^ curves and explain how the temporal filter and the dark count of the single-photon detector affect the single-photon purity. A typical PL decay curve comprises both biexciton emission and electron–hole (exciton) emission. The PL decay rate of the biexcitons is larger than that of the excitons because of additional recombination processes from nonradiative Auger recombination and an increase in the number of radiative recombination path^40,41^. With the use of a temporal filter, the remaining PL decay curve is dominated by exciton emission. With no temporal filter, the g^(2)^ curve shows the signal originating from the STPE process of the biexcitons at the time origin. With the temporal filter applied, some of this center peak is removed, as shown in Fig. 1c. The remaining signal at the g^(2)^ time origin arises mainly from the start-stop event composed of PL signal and dark count of the single-photon detector. The aim of the use of SSPDs with suppressed dark counts is to reduce this remaining signal at the time origin.

In Fig. 1d,e, the experimental data of PL decay and g^(2)^ curves obtained on CdSe/ZnS core-shell CQDs that have PL peak wavelength at 655 nm (Qdot655, ThermoFisher; Sample A) with and without the application of the temporal filter are plotted on logarithmic scales. The fast and slow decay components in the decay curve are the PL of biexcitons and those of excitons, respectively. We do not need to consider contributions of PL from other multiexcitons, such as charged excitons and charged biexcitons, for the interpretation of the decay curve. This is because we extracted the PL signal in the time periods having high PL intensity and long PL lifetime, which correspond to neutral condition, by measuring the time-dependent PL fluctuation with binning time of 50 ms, as explained in our previous papers^23,40^. From the decay rate of the slow and fast PL decay components in Fig. 1d, the PL lifetimes of excitons (${\tau }_{{\rm{X}}}$) and biexcitons (${\tau }_{{\rm{XX}}}$) are determined as 30 ns and 1.5 ns, respectively. To remove the PL of the biexcitons, the width of the temporal filter (δ) is set to 12 ns, which is much longer than ${\tau }_{{\rm{XX}}}$. By applying the temporal filter, the height of the g^(2)^ side peak is decreased from 10000 to 4000 counts, while that of the g^(2)^ center signal is decreased from 2000 to 60 counts. Through the use of the temporal filter, the line shape of g^(2)^ side peaks shows no large change. However, the g^(2)^ line shape around the time origin changes significantly, characterized by the transformation from a peak structure to a clear dip structure.

### Importance of g^(2)^ dip structure for high-performance SPSs

**Figure 2:**
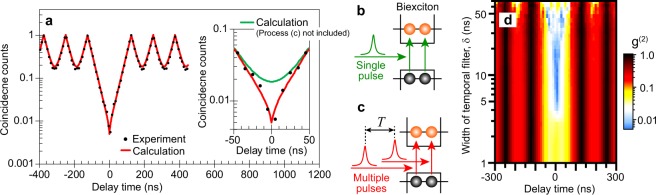
g^(2)^ dip structure representing the biexciton creation process. (**a**) Experimental result of g^(2)^ obtained from Sample B with a temporal filter (black dots) and theoretical calculation (red line) fitted to the experimental data. The inset shows an enlargement of the plots around the time origin. The green line is a theoretical calculation excluding the biexciton creation process through double-pulse excitation. (**b**) Biexciton creation process through single-pulse excitation. (**c**) Biexciton creation process through double-pulse excitation. (**d**) A color map of g^(2)^ data plotted as functions of delay time and width of temporal filter (δ). The value of g^(2)^(0) becomes less than 0.01 for 5 ns < δ  < 20 ns.

The appearance of the characteristic dip structure in the g^(2)^ curve at the time origin is undoubtedly necessary for the realization of high single-photon purity. Here, we compare the experimental g^(2)^ data with theoretical calculations to investigate the mechanism of the appearance of the dip structure and its effect on the single-photon purity. Figure 2a shows the g^(2)^ data obtained after applying the temporal filter. This data was obtained on CdSe/ZnS core-shell CQDs that have PL peak wavelength at 620 nm (Sigma-Aldrich; Sample B). The width of the temporal filter δ is set to 10 ns; the δ dependence of the g^(2)^ data are shown in Fig. 2d as a color map, where the g^(2)^ values are shown as functions of the delay time and δ. The red curve in Fig. 2a is a result of the theoretical calculation fitted to the experimental data. The calculation was performed using the following equation:

1 $${g}^{(2)}(t)={A}_{0}+{B}_{0}\cdot \exp (-\frac{|t|}{{\tau }_{{\rm{X}}}})+\sum _{n\ne 0}{B}_{n}\cdot \exp (-\frac{|t-n\cdot T|}{{\tau }_{{\rm{X}}}})\cdot [1-\exp (-\frac{|t|}{{\tau }_{{\rm{X}}}})],$$

where *A*_0_ is a constant value for the background offset, *B*_0_ is the height of the center peak, *B*_*n*_ is the height of the side peaks, *n* is the position of the side peaks, *τ*_x_ is the PL lifetime of excitons, and *T* is the time interval between successive excitation pulses. The second term corresponds to the signal originating from the STPE process of the biexcitons, while the third term corresponds to the coincidence counts originating from two photons emitted from two different excitons. The third term does not include the contribution for *n* = 0, because two different excitons cannot be created by a single excitation pulse, i.e., the creation of two excitons with single pulse results in the formation of biexcitons, as shown in Fig. 2b. Additionally, the third term is multiplied by a factor $[1-\exp (-|t|/{\tau }_{{\rm{X}}})]$ considering the fact that two different excitons cannot be created in a single quantum dot, even if they are excited by two different pulses^44^. In our case, if an exciton created by the first pulse does not recombine until the next pulse hit the sample, the creation of one more exciton by the second pulse results in the formation of a biexciton, as shown in Fig. 2c.

In the fitting procedure, *B*_0_ is considered to be zero, because the use of the temporal filter reduces the contribution of the signal originating from the STPE process of the biexcitons. In addition, the value of *τ*_X_ is fixed at 25 ns, since *τ*_X_ can be determined from PL decay curve. We consider the same value of *B*_*n*_ (=*B*_1_) for all *n*. We assume that the effects of temporal filter to the function form are negligible for the condition of δ ≪ *T*. Under these conditions, the fitting parameter is only the ratio between *A*_0_ and *B*_1_. In the present case, *A*_0_/*B*_1_ = 0.005. In the inset of Fig. 2a, the enlarged plot for the small-delay-time region is shown. The green line indicates the theoretical curve where the last term $[1-\exp (-|t|/{\tau }_{{\rm{X}}})]$ is replaced with a time-independent constant. The calculation including the last term agrees well with the experimental data. This suggests that the use of temporal filter can remove signals originating from the STPE from biexcitons created by two successive excitation pulses.

The color map in Fig. 2d, where the experimental data of g^(2)^ values are shown as functions of the delay time and δ, indicates that g^(2)^(0) can be <0.01 even for δ ~ 5 ns. For the present experimental condition, where the interval time between successive laser pulses is 125 ns and the exciton PL lifetime is 30 ns, the condition of δ ~ 5 ns does not cause a large reduction of the single-photon generation rate. In fact, the reduction in the single-photon generation rate, which is ~100 kHz in the present experimental condition, is only ~15% at δ ~ 5 ns. Furthermore, by choosing a sample whose biexciton PL lifetime is much shorter than the present experiment, the value of δ can be further reduced. For example, using CQDs whose biexciton lifetime is ~40 ps^45^, high single-photon purity can be achieved for δ < 100 ps, thus allowing an increase in the excitation frequency. Such increase in the excitation frequency is desirable for realizing high single-photon generation rates. As we have indicated in the present experiment, even for high-frequency excitation conditions, temporal filtering for single-photon purification is available because it can remove signals originating from the STPE from biexcitons created by not only single pulse but also successive multiple excitation pulses. Therefore, by using the temporal filter under high-frequency excitation conditions, high single-photon purity and high photon-generation rates can be simultaneously achieved.

### Observation of ultrahigh single-photon purity and comparison with other SPSs

**Figure 3:**
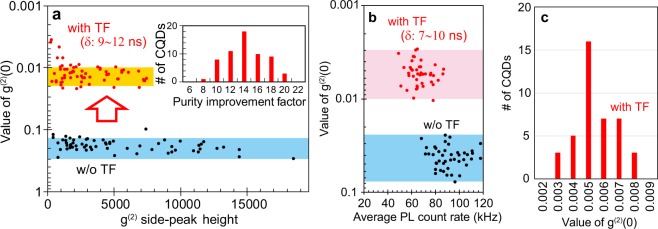
Results of single-photon purities. (**a**) The value of g^(2)^(0) obtained from Sample A plotted as a function of the g^(2)^ side-peak height. The data without and with applications of temporal filters (TF) are plotted with black and red dots, respectively. The inset shows the number of CQDs plotted as a function of the purity improvement factor, defined as the ratio between g^(2)^(0) values before and after the application of the temporal filter. (**b**) The values of g^(2)^(0) obtained on Sample B. The results obtained with and without TF are plotted as a function of average PL count rates. (**c**) Number of CQDs plotted as a function of the value of g^(2)^(0) obtained with TF.

Next, we demonstrate the impact of the temporal filter on the values of g^(2)^(0). Figure 3a plots the values of g^(2)^(0) obtained on Sample A for 63 CQDs as a function of the g^(2)^ side-peak height. The black dots are the results obtained without the temporal filters. They are located in the blue bar that indicates the g^(2)^(0) range from 0.14 to 0.3. The red dots are the results after applications of the temporal filters, where the values of δ were set between 9 ns and 12 ns to obtain the minimum values of g^(2)^(0) for each CQDs. They are located in the orange bar that corresponds to g^(2)^(0) between 0.01 and 0.02. In the inset of Fig. 3a, the number of CQDs is plotted as a function of the purity improvement factor, defined as the ratio between g^(2)^(0) values before and after the application of temporal filtering. The values range from 8 to 20. In Fig. 3b, the results of g^(2)^(0) obtained on 43 CQDs for Sample B are plotted as a function of average PL count rates. The black and red dots correspond to the data without and with the use of temporal filters, respectively. The values of δ for obtaining the minimum values of g^(2)^(0) on each CQDs were ranging from 7 ns to 10 ns. Figure 3c shows the number of CQDs as a function of g^(2)^(0) value obtained with the use of temporal filter. More than 95% CQDs appear in the range of *g*^(2)^ (0) = 0.005 ± 0.002. From this result, we conclude that we observed the ultrahigh single-photon purity corresponding to g^(2)^(0) = 0.005.

**Figure 4:**
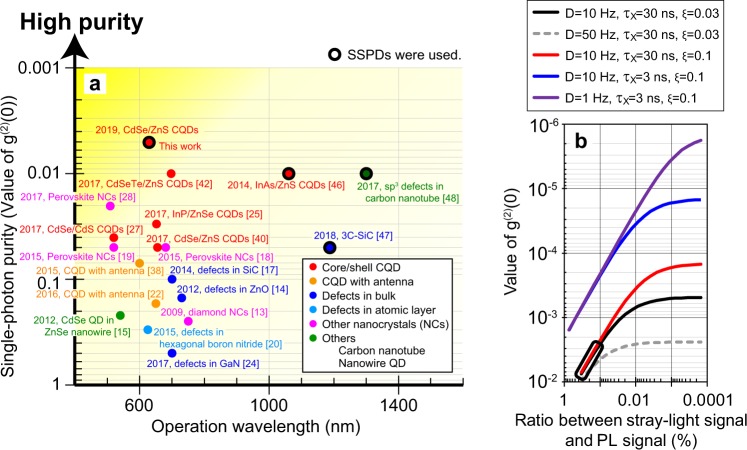
Present situation and future improvements of single-photon purities. (**a**) Single-photon purities reported for room-temperature SPSs comprising various materials. The results are plotted as a function of the operation wavelength. The solid circle indicates the use of superconducting-nanowire single-photon detectors (SSPDs). (**b**) Expected values of g^(2)^(0) calculated for the various experimental conditions considering the changes of the stray-light signal, the dark count of single-photon detector (D), the PL lifetimes of excitons (*τ*_*X*_), and the detection efficiency (*ξ*). The black rectangle at the bottom, left corner indicates the present situation of our experiments.

Here we compare the above result with those reported by other groups. Figure 4a summarizes the single-photon purities reported for room-temperature SPSs composed of various materials such as core/shell CQDs^25,27,40,42,46^, CQDs with antenna structures^22,38^, defects in bulk crystals^14,17,24,47^, defects in atomic layers^20^, perovskite nanocrystals^18,19,28^, carbon nanotubes^48^, diamond nanocrystals^13^, and nanowire quantum dots^15^. While many of these earlier works utilized avalanche photodiodes as single-photon detectors, three papers reported the use of SSPDs^46–48^. Two of these earlier works, studying InAs/ZnS CQDs^46^ and defects in carbon nanotubes^48^, reported good single-photon purities showing g^(2)^(0)~0.01. However, in their work, the SSPDs were used to detect PL in the short-wave infrared region and telecom wavelengths in the infrared region. Meanwhile, our SSPDs are designed to have high visible-region detection efficiency^43^. With this SSPD, we achieve the observation of the ultrahigh single-photon purity showing g^(2)^(0) = 0.005. We believe that the use of SSPD and temporal filter are indispensable to achieve further improvement of single-photon purity on room-temperature SPSs comprising not only CQDs but also various materials and devices.

Finally, we present results of numerical calculations of g^(2)^(0) and discuss the possibility to realize further improvements in single-photon purity. Figure 4b shows the calculated values of g^(2)^(0) as a function of the ratio between the stray-light signal and the PL signal ( = C). A detailed explanation of the calculation model is provided in Supplementary information. In the present experiments, photons emitted from the glass substrate, which has nearly time-independent profile, remained as the stray-light signal. The black line shows g^(2)^(0) for dark count rate of SSPDs (D) = 10 Hz, *τ*_X_ = 30 ns, and the detection efficiency for one side of Hanbury Brown-Twiss (HBT) interferometer (*ξ*) = 0.03. The present situation of our experiment is indicated by a black rectangle. The calculated results indicate that the decrease in C causes the reduction of g^(2)^(0) to <10^−3^. This small value of g^(2)^(0) is hard to realize if the detector has large dark counts (~50 Hz) as shown by broken grey line. The red line shows the results calculated for similar parameters, except for a larger assumed *ξ* of 0.1. For small C of <0.001%, the increase of *ξ* from 0.03 to 0.1 yields in smaller values of g^(2)^(0) approaching 10^−4^. The blue line shows the calculated results with D = 10 Hz, *τ*_X_ = 3 ns, and *ξ* = 0.1. The shorter exciton lifetime yields an increase in the g^(2)^ side-peak height, allowing improved purity. For this condition, especially for small values of C of <0.001%, the reduction of D to 1 Hz yields an extremely low g^(2)^(0) of <10^−5^, as shown by the purple line. This result suggests that the ultralow-dark-count detection of temporally purified single photons will enable confirmation of the ultrahigh performance of room-temperature SPSs showing g^(2)^(0) of <10^−5^.

## Discussions

In conclusion, we revealed the superior properties of CQD emitters, including high single-photon purity and high single-photon generation rate, based on the use of SSPDs and temporal filtering. We achieved observation of g^(2)^(0) = 0.005, which is the best recorded purity for room-temperature SPSs comprising various nanomaterials and devices. We demonstrated numerically that g^(2)^(0) of <10^−5^ can be realized by optimizing the experimental conditions. These findings are strong evidence of the attractiveness of CQDs as candidates for room-temperature SPSs, which may be fundamental components of quantum technologies. High-performance SSPDs in the visible region, in conjunction with temporal filtering, will be indispensable tools for the study of superior properties in room-temperature SPSs comprising various materials^13–29^. We believe that further investigations, including further single-photon purification, increases in single-photon generation rate, and improved efficiency in the generation of indistinguishable photons at room temperature, are important tasks for accelerating the various applications of quantum technologies^7,49^.

## Methods

### Details of experimental setup

The setup for the optical measurement is shown in Supplementary Fig. 1. Single photons were generated from CQDs drop-casted on a cover slip. We employed various types of CQDs with core/shell structures, such as CdSe/ZnS CQDs. The data shown in the present paper are high-quality results obtained from two kinds of CdSe/ZnS CQDs samples with PL at 655 nm (Qdot655 purchased from ThermoFisher Scientific; Sample A) and 620 nm (purchased from Sigma-Aldrich; Sample B). As to the Sample A, we have studied their size distribution, Auger rate, and radiative lifetime in the early works^23,40^. The CQD samples were diluted in toluene solution with a polymer (Polymethyl methacrylate) and dropcasted on cover glass (Matsunami). The optical excitation of the CQDs was performed with a picosecond laser operating with a frequency of 8 MHz at 485 nm, obtained by up-converting the 970 nm output of a Ti:Sapphire laser (Tsunami; Spectra-Physics) by the second harmonic generation. While we have reported results of similar experiments recently^50^, the studies of g^(2)^ lineshape under 8 MHz on both sample A and B, which are essential points for the findings reported in the present paper, were performed for the first time in the present work. The excitation laser power was set to be 20–50 W/cm^2^ to realize mean exciton population around 0.2–0.5. The single photons emitted from the single CQDs were efficiently collected using an oil-immersion lens with the numerical aperture of 1.4. With this oil-immersion lens, which is not available at cryo-temperature condition, one can easily achieve high single-photon generation rate without relying on utilizing antenna structures. A confocal microscope and HBT interferometer were used to measure the g^(2)^ of the emission from the single CQDs.

### Details of SSPD and detection wavelength

The single photons were detected by two SSPDs connected to the optical fibers. The SSPDs were designed to have high detection efficiency from 450 nm to 600 nm. The designed and measured properties of spectral response and detection efficiency were provided in our previous paper^43^. The detection efficiency is designed to be above 70% for 600 nm. Their dark counts were 5–10 Hz and their temporal resolution were 60 ps. We used multiple long pass and short pass filters (High performance OD4 filters; Edmund Optics) to selectively detect PL from single CQDs. For Sample A, we used three long pass filters at 625 nm and two short pass filters at 675 nm. For Sample B, we used three long pass filters at 600 nm and two short pass filters at 650 nm. This difference of detection wavelength caused the change in the amount of stray-light signal coming from glass substrate. We believe that this change of background stray light is the origin of the difference in the data quality between Sample A and Sample B.

### Data recording and analysis

The arrival times of the single photons were recorded on a time-correlated single-photon counting (TCSPC) board (TimeHarp 260 nano dual; Pico Quant). The TCSPC board was operated under a time-tagged time-resolved mode, where all single-photon arrival times were recorded in a text file. For each measurement of single CQDs, the total integration time was set to 200 s. The recorded single-photon detection signals were separated with the binning time of 50 ms into 4000 sections, from which we analyzed 4000 time-dependent PL decay and g^(2)^ curves. The PL lifetimes of the PL decay curves in each binning time were determined by performing theoretical fitting of the data using single-exponential functions. Considering the changes in PL lifetimes and intensities, the 4000 sections were separated into two categories: signals obtained for neutral versus ionized conditions. The former is characterized by high PL intensity and long PL lifetime, while the latter is characterized by low PL intensity and short PL lifetime^23,40^. In the present paper, we focused on the high-quality data obtained for the neutral conditions.

## Supplementary information

Supplementary Information
