# Supplementary material for: Superior properties in room-temperature colloidal-dot quantum emitters revealed by ultralow-dark-count detections of temporally-purified single photons
Source: Sci Rep. 2019 Nov 4;9:15941. doi: 10.1038/s41598-019-52377-1 (PMC6828765; doi:10.1038/s41598-019-52377-1)
Supplement: Supplementary file 1 — Supplementary Information [file 41598_2019_52377_MOESM1_ESM.pdf]

Supplementary Information for
Superior properties in room-temperature colloidal-dot quantum emitters revealed by ultralow-dark-count detections of temporally-purified single photons

Toshiyuki Ihara, Shigehito Miki, Toshiki Yamada, Takahiro Kaji, Akira Otomo, Iwao Hosako, and Hirotaka Terai*

*Corresponding author. Email: t-ihara@nict.go.jp

This file includes:
Supplementary Text
Figure S1

Supplementary Text

A model to calculate $g^{(2)}(0)$ under various experimental conditions

When only exciton emission is considered, the height of the $g^{(2)}$ side peak can be written in the following form:

$$g_S^{(2)}(t) = T_{accum} \cdot \delta t \cdot F \cdot \{P_X \cdot \eta_X\}^2 \cdot \xi_A \cdot \xi_B \cdot \sum_{n \neq 0} \frac{1}{2\tau_X} \cdot \exp\left(-\frac{|t - n \cdot T|}{\tau_X}\right) \cdot \left[1 - \exp\left(-\frac{|t|}{\tau_X}\right)\right], \qquad \text{(S1)}$$

where T_{accum} is the accumulated time, δt is the interval of time axis, F is the repetition rate, P_X is the excitation probability of the excitons, η_X is the quantum efficiency of excitons, ξ_A (ξ_B) is the detection efficiency of detector A (B), τ_X is the PL lifetime of excitons, and n is the position of the side peaks. When a Poisson distribution, $P(\langle N \rangle, m) = \langle N \rangle^m \exp(-\langle N \rangle)/m!$, is taken into account for the photons in the incident light pulse, the excitation probabilities of excitons can be written in the form of $P_X = \sum_{m=1}^{\infty} P(\langle N \rangle, m) = 1 - \exp(-\langle N \rangle)$. The height of the $g^{(2)}$ side peak ($g_{S,\mathrm{top}}^{(2)}$) can be calculated by applying $t = n \cdot T$ in the eq. (S1) for the condition of $t \gg \tau_X$. This results in the following form;

$$g_{S,\mathrm{top}}^{(2)} = T_{accum} \cdot \delta t \cdot F \cdot {P_X}^2 \cdot {\eta_X}^2 \cdot \xi_A \cdot \xi_B \cdot \frac{1}{2\tau_X}. \qquad \text{(S2)}$$

On the other hand, the $g^{(2)}$ background signal is written in the following form;

$$g_{bg}^{(2)} = 2 \cdot T_{accum} \cdot \delta t \cdot [F \cdot P_X \cdot \eta_X \cdot \{C_1 \cdot F \cdot \langle N \rangle \cdot \eta_X \cdot 2 \cdot \xi_A \cdot \xi_B + C_2 \cdot (\xi_A + \xi_B)\}], \qquad \text{(S3)}$$

where C_1 is ratio between stray-light signal and PL signal, and C_2 is the detector dark count. Here we assumed the intensity of PL signal is written by $F \cdot \langle N \rangle \cdot \eta_X \cdot \xi_A$ or by $F \cdot \langle N \rangle \cdot \eta_X \cdot \xi_B$ for detector A and B, respectively. The value of $g^{(2)}(0)$, which is determined by the ratio between $g_{S,\mathrm{top}}^{(2)}$ and $g_{bg}^{(2)}$, can be written in the following form;

$$g_0^{(2)} = \frac{g_{bg}^{(2)}}{g_{S,\mathrm{top}}^{(2)}} = \frac{4\tau_X\{C_1 \cdot F \cdot \langle N \rangle \cdot \eta_X \cdot 2 \cdot \xi_A \cdot \xi_B + C_2 \cdot (\xi_A + \xi_B)\}}{P_X \cdot \eta_X \cdot \xi_A \cdot \xi_B}. \qquad \text{(S4)}$$

This equation (S4) was used to obtain the calculation results shown in Fig. 4b.

Supplementary Figure

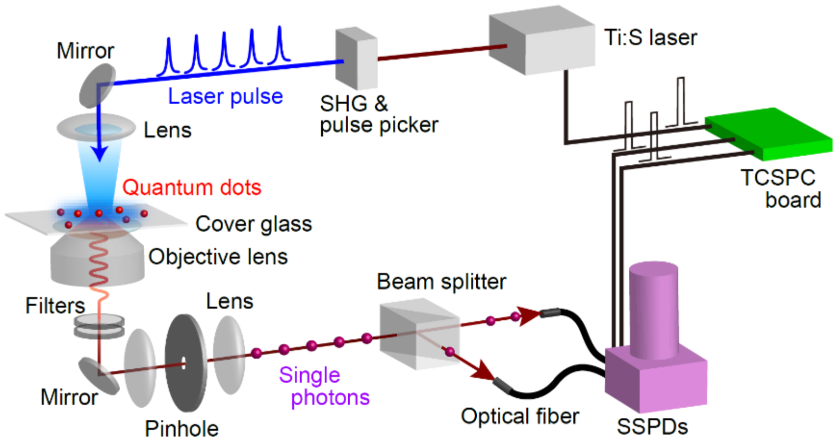

Fig. S1 A schematic drawing of the experimental setup. The single photons emitted from single quantum dots were collected by an oil-immersion objective lens and spatially purified by a pinhole located at the confocal position. The single photons were detected using superconducting nanowire single-photon detectors (SSPDs). The temporal purification of the single photons was performed on the data analysis of the signals recorded using a Time-correlated single-photon counting (TCSPC) board.
